# Molecular Identification of *Toxoplasma gondii* Isolates From Spontaneous Abortion Placentas in Women in Eastern Iran

**DOI:** 10.1155/japr/4639653

**Published:** 2026-05-19

**Authors:** Peyman Bagherzadeh, Seyed Mohammad Mousavi, Amir Tavakoli Kareshk, Mahmoodreza Behravan, Davod Javanmard

**Affiliations:** ^1^ Infectious Diseases Research Center, Birjand University of Medical Sciences, Birjand, Iran, bums.ac.ir; ^2^ Research Center for Hydatid Disease in Iran, Institute of Infectious Diseases and Tropical Medicine, Department of Medical Parasitology, Afzalipour School of Medicine, Kerman University of Medical Sciences, Kerman, Iran, kmu.ac.ir; ^3^ Student Research Committee, Birjand University of Medical Sciences, Birjand, Iran, bums.ac.ir

**Keywords:** B1 gene, nested PCR, spontaneous abortion, *Toxoplasma gondii*

## Abstract

**Background:**

*Toxoplasma gondii* is a globally prevalent protozoan parasite associated with adverse pregnancy outcomes, including miscarriage and congenital abnormalities. This study investigated the molecular presence of *T. gondii* in placental tissues from women with spontaneous abortion and explored potential associated risk factors in Birjand, Eastern Iran.

**Materials and Methods:**

A total of 100 placental or fetal tissue samples were collected from women with confirmed spontaneous abortion during 2022–2023. Genomic DNA was extracted and analyzed using nested PCR targeting the B1 gene of *T. gondii*. Demographic, obstetric, and behavioral data were also collected and statistically analyzed.

**Results:**

*T. gondii* DNA was detected in 8% of samples. A significant association was observed between infection and contact with domestic animals, including pet care (*p* < 0.05). No significant relationships were identified with maternal age, place of residence, or prior abortion history (*p* > 0.05). Notably, all positive cases were identified in pregnancies beyond 8 weeks of gestation (*p* < 0.05), suggesting an increased detectability or susceptibility in later gestational stages.

**Conclusion:**

These findings indicate that *T. gondii* infection may contribute to a subset of spontaneous abortions in this region. The results highlight the importance of targeted preventive strategies, including improved hygiene practices and awareness regarding animal exposure. Further large‐scale studies integrating both molecular and serological approaches are warranted to better elucidate the role of *T. gondii* in adverse pregnancy outcomes.

## 1. Introduction

*Toxoplasma gondii* is an obligate intracellular protozoan parasite capable of infecting virtually all warm‐blooded animals, including humans. Its global significance stems from a combination of factors: First, it can establish long‐term latent infections in host tissues; second, it is highly prevalent in many regions; and third, it poses a particular threat to immunocompromised individuals and developing fetuses [1, 2]. Transmission most commonly occurs through ingesting raw or undercooked meat containing tissue cysts, or via food, water, and soil contaminated with oocysts shed in feline feces. Additionally, less common but still recognized routes include organ transplantation from infected donors, blood transfusions containing parasitic stages, and vertical transmission from mother to fetus when a nonimmune woman acquires the infection during pregnancy. While immunocompetent individuals may display mild or no symptoms, *T. gondii* can cause severe or life‐threatening illness in immunocompromised patients [1, 3, 4]. The worldwide prevalence of toxoplasmosis varies widely depending on climate, regional dietary customs, and socioeconomic conditions. In areas where residents habitually consume undercooked meats (e.g., parts of South America and Europe), seroprevalence rates can be as high as 50%–80% [2, 5]. Socioeconomic challenges such as limited access to clean water or insufficient public health infrastructure may further increase exposure risk by reducing the availability of properly treated foods and by fostering closer contact with free‐roaming cats that can shed oocysts. Consequently, understanding local risk factors is vital to developing effective preventive measures, including public health education, safe meat‐handling practices, and consistent screening strategies in at‐risk populations. *T. gondii* infection is often mild in immunocompetent individuals; it poses a significant threat to immunocompromised patients and developing fetuses [6]. In pregnant women who acquire a primary infection, the parasite can be transmitted across the placenta, leading to devastating outcomes such as miscarriage, stillbirth, or congenital disease marked by hydrocephalus, chorioretinitis, and neurological impairment [2]. Such serious outcomes underscore the need for early diagnosis and careful monitoring during gestation to reduce vertical transmission risk. Although infections like toxoplasmosis have been implicated in spontaneous abortion, the direct association remains contested; some observational studies report a link between *T. gondii* seropositivity and higher abortion rates, whereas others find no significant correlation [6–8]. This incongruity in findings points to the need for larger scale, well‐controlled studies encompassing both serological markers (e.g., IgG and IgM) and molecular methods (e.g., PCR) to clarify *T. gondii*′*s* precise contribution to spontaneous abortion risk [2].

This research gap is particularly pronounced in areas like Birjand in Eastern Iran, where detailed data on the molecular prevalence of *T. gondii* among women with spontaneous abortion are scarce. Birjand′s arid climate is known to limit the environmental survival of *T. gondii* oocysts, yet local dietary customs, which include the consumption of grilled meats that may be undercooked, could still pose a transmission risk. Coupled with an unknown prevalence of the parasite in the local stray cat population, these factors create a unique epidemiological context. Investigating the prevalence in Birjand is therefore critical to understanding the transmission dynamics in this specific environment. Clarifying the role of the parasite in this population is critical for public health, as early detection through targeted screening can enable timely interventions, reduce the risk of congenital infection, and ultimately lower healthcare costs associated with long‐term complications [7, 8]. Therefore, the primary objective of the current study is to determine the molecular prevalence of *T. gondii* in tissue samples from women with spontaneous abortion in Birjand. A secondary aim is to investigate potential associations between infection status and various demographic, obstetric, and behavioral risk factors to better inform regional prevention strategies.

## 2. Material and Methods

### 2.1. Study Design

A descriptive cross‐sectional study was conducted after receiving approval from the institutional ethics committee. Participants were selected by convenience sampling from women presenting to the obstetrics ward of Valiasr Hospital in Birjand during 2022–2023.

### 2.2. Sample Size

A total of 100 women were included. A convenience (in‐clinic) sampling was employed. Inclusion criteria required participants to be Iranian nationals with a health file for prenatal care and a current spontaneous abortion. The primary exclusion criterion was the presence of any known underlying disease that could act as a confounding factor for spontaneous abortion. Specifically, women with chronic conditions such as diabetes mellitus, uncontrolled hypertension, autoimmune disorders (e.g., systemic lupus erythematosus), or other known chronic infectious diseases (e.g., HIV or hepatitis) were excluded from the study. Participants with non‐Iranian nationality were also excluded.

### 2.3. Data Collection Procedures

Data were obtained using a standardized questionnaire administered by trained researchers or through a structured interview form. Demographic variables included participants′ age, categorized as younger than 20, 20–29, 30–40, or over 40 years, as well as their area of residency (urban or rural). Obstetric data encompassed the number of pregnancies, gestational age, any previous spontaneous abortion(s), and the occurrence of other obstetric complications such as premature rupture of membranes and preterm labor. Additional details pertaining to participants′ medical history, prenatal care, and recognized risk factors (e.g., contact with animals such as cats, or the consumption of raw or undercooked meats) were also collected.

### 2.4. Tissue Sampling

Immediately following confirmation of spontaneous abortion, a placental or fetal tissue sample was collected. The portion of tissue was typically obtained from the central region of the maternal side of the placenta, when available, and subsequently placed in sterile tubes. To ensure preservation of DNA integrity, samples were initially stored at −20°C, followed by transfer to −70°C for long‐term storage.

### 2.5. Laboratory Analysis

Genomic DNA was extracted from all placental specimens using a combination of a commercial tissue DNA purification kit (FavorGen) and manual phenol–chloroform steps to ensure both purity and adequacy of yield; the quality and concentration of extracted DNA were verified spectrophotometrically. Molecular detection of *T. gondii* was performed using a nested PCR targeting the B1 gene, which has approximately 35 copies in the parasite genome. In the first amplification, two primers (s1: 5 ^′^‐TGTTCTGTCCTATCGCAACG‐3 ^′^ and As1: 5 ^′^‐ACGGATGCAGTTCCTTTCTG‐3 ^′^) were used to generate an approximately 580‐bp product. The second (nested) amplification employed the primers s2: 5 ^′^‐TCTTCCCACAGTTCCTTTCTG‐3 ^′^ and As2: 5 ^′^‐CTCGACAATACGCTGCTTGA‐3 ^′^, yielding a final PCR amplicon of around 530 bp. The thermal cycling conditions for the first PCR round were as follows: an initial denaturation at 95°C for 5 min; followed by 35 cycles of denaturation at 94°C for 30 s, annealing at 58°C for 45 s, and extension at 72°C for 1 min; with a final extension at 72°C for 7 min. For the nested (second) round, 2 *μ*L of the first‐round product was used as a template. The cycling conditions were as follows: an initial denaturation at 95°C for 5 min; followed by 35 cycles of denaturation at 94°C for 30 s, annealing at 60°C for 30 s, and extension at 72°C for 45 s; with a final extension at 72°C for 7 min. Each reaction was run in a standard thermal cycler (Analytic Gena, Germany) according to the recommended cycling protocol for nested amplification of the B1 locus. For each batch of samples, positive control DNA (from a known *T. gondii* strain) and a nuclease‐free water negative control were included.

### 2.6. Statistical Analysis

Data collected through questionnaires, interviews, and molecular assays were entered into SPSS software for analysis. The chi‐square test (or Fisher′s exact test where appropriate) was used to explore associations between *T. gondii* positivity and clinical or demographic parameters. A *p* value < 0.05 was considered statistically significant. All data were handled confidentially, and participants retained the right to withdraw from the study at any time.

## 3. Results

**Figure 1:**
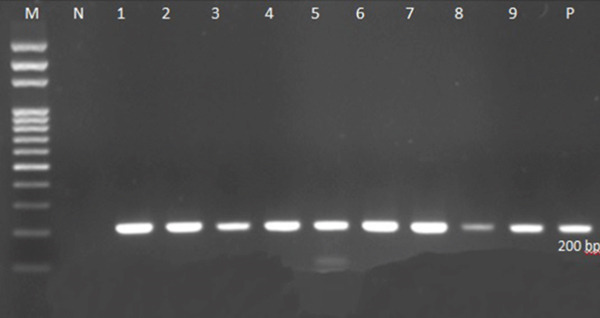
PCR product electrophoresis on 1.5% agarose gel for *Toxoplasma gondii* B1 gene. Lines 1–9: PCR product obtained from B1 gene amplification; Line M: 100 bp marker; Line N: negative control; Line P: positive control.

A total of 100 women with spontaneous abortion were included in the study. The mean maternal age in this cohort was approximately 31.6 years, with most participants (49%) falling within the 30–40‐year age group, and the lowest frequency occurring among those above 40 years old (9%) and under 20 years old (6%) (Table 1). No statistically significant association was noted between age and *T. gondii* positivity (*p* > 0.05). In terms of residence, 68% of participants lived in rural areas, whereas 32% resided in urban settings. However, the distribution of *T. gondii* infection did not differ significantly based on place of residence (Table 1) (*p* > 0.05). PCR analysis targeting the B1 gene revealed that 8 out of 100 tissue samples (8%) were positive for *T. gondii*, whereas 92 (92%) were negative. Figure 1 (electrophoresis on 1.5% agarose gel) confirmed the presence of a 200‐bp amplicon in positive cases, consistent with *T. gondii* DNA. Gestational age ranged from under 8 weeks up to 20 weeks. A total of 62 (62%) participants were between 8 and 20 weeks of gestation, whereas 38 (38%) were under 8 weeks. Notably, all PCR‐positive cases occurred in pregnancies exceeding 8 weeks (*p* < 0.05), indicating a significant association between later gestational weeks and infection detection (Table 1). Of the study participants, 38% reported a history of one or more previous abortions, but this variable showed no statistically significant relationship with *T. gondii* positivity (*p* > 0.05). Similarly, the number of prior pregnancies (range of one to seven) did not show any significant correlation with PCR positivity. Regarding preterm birth, only 9% of participants reported a history of preterm delivery. None of these individuals tested positive for *T. gondii*, and no significant association was detected between preterm delivery history and current *T. gondii* infection status (*p* > 0.05) (Table 2). None of the participants reported active smoking or a notable history of intravenous drug use or criminal conviction. In total, 23% of participants reported contact with domestic animals, and 4% owned or cared for a household pet (typically cats). In contrast to other variables, both history of contact with domestic animals and pet care showed a statistically significant association with *T. gondii* positivity (*p* < 0.05) (Table 1). None of the participants reported smoking or intravenous drug use, and these factors, along with having tattoos or a criminal record, showed no correlation with infection status (*p* > 0.05) (Table 2). Among the 100 participants, 14% tested positive for human papillomavirus (HPV) via PCR. Nevertheless, no significant overlap or interaction between HPV carrier state and *T. gondii* infection was observed (*p* > 0.05). With respect to mode of conception, 97% became pregnant naturally and 3% via in vitro fertilization (IVF). PCR positivity did not differ by method of conception (*p* > 0.05) (Table 2).

**Table 1:** Demographic and clinical characteristics of women with spontaneous abortion.

Demographic characteristics		No.	(%)	*Toxoplasma gondii* positive no.	*Toxoplasma gondii* positive %	*p* value
Age	< 20	6	6	0	0	> 0.05
	20–29	36	36	3	8.3	
	30–40	49	49	5	10.2	
	> 40	9	9	0	0	

Location	Rural	68	68	2	2.9	> 0.05
	Urban	32	32	6	18.7	

Job	Housewife	77	77	6	7.8	> 0.05
	Employee	17	17	1	5.9	
	Student	2	2	0	0	
	Educated person	2	2	0	0	
	Self‐employment	2	2	1	50	

Monthly income	Under 3 million	35	35	5	14.3	> 0.05
	3–5 million	43	43	2	4.6	
	5–7 million	16	16	1	6.3	
	Above 7 million	6	6	0	0	

Gestational age	Under 8 weeks	38	38	0	0	< 0.05
	8–20 weeks	62	62	8	12.9	

History of contact with domestic animals	Yes	23	23	6	26.1	< 0.05
	No	77	77	2	2.6	

Pet care	Yes	4	4	4	100	< 0.05
	No	96	96	4	4.2	

Smoking	Yes	0	0	0	0	> 0.05
	No	100	100	8	8	

History of intravenous injection	Yes	0	0	0	0	> 0.05
	No	100	100	8	8	

Tattoo	Yes	0	0	0	0	> 0.05
	No	100	100	8	8	

Criminal case	Yes	0	0	0	0	> 0.05
	No	100	100	8	8	

**Table 2:** Pregnancy and abortion characteristics in women with spontaneous abortion.

Pregnancy and abortion characteristics		No.	(%)	*Toxoplasma gondii* positive no.	*Toxoplasma gondii* positive %	*p* value
Premature rupture of membranes (PROM)	Positive	4	4	1	25	> 0.05
	Negative	96	96	7	7.3	

Number of childbirth	1	23	23	0	0	> 0.05
	2	17	17	0	0	
	3	26	26	0	0	
	4	15	15	1	6.7	
	5	14	14	3	21.4	
	6	3	3	2	66.7	
	7	2	2	2	100	

History of abortion	Yes	38	38	0	0	> 0.05
	No	62	62	8	12.9	

Contraceptive methods	No contraception	83	83	7	8.4	> 0.05
	LD pill	8	8	1	12.5	
	Condom	4	4	0	0	
	Injection	4	4	0	0	
	Natural	1	1	0	0	

History of preterm childbirth	Yes	9	9	0	0	> 0.05
	No	91	91	8	8.8	

Type of pregnancy	Natural	97	97	8	8.2	> 0.05
	IVF	3	3	0	0	

## 4. Discussion

In the present study, 8% of the examined tissue samples from women with spontaneous abortion were positive for *T. gondii* using molecular (PCR) techniques. Although the overall proportion of positive samples was relatively low, this finding reinforces the importance of considering toxoplasmosis as one potential cause of reproductive complications, especially in women of childbearing age. Of note, a significant association was detected between *Toxoplasma* positivity and contact with domestic animals, including pet care (*p* < 0.05). This finding highlights a key risk factor in our study population, likely linked to environmental exposure to oocysts shed in feline feces. In contrast, no significant association was detected for other factors like age, prior history of preterm birth, smoking status, or place of residence (urban vs. rural). The lack of correlation with these other variables suggests that while broader environmental and behavioral patterns are important, direct contact with potential host animals is a primary predictor of infection in this cohort [9, 10]. The prevalence rates of toxoplasmosis in pregnant women and women with spontaneous abortion differ widely in various parts of the world. Investigations in Northern European countries such as Norway, Finland, and Denmark have shown prevalence rates typically ranging from about 10% to nearly 30% [11–14]. Within Iran, some provincial reports have demonstrated even higher seropositivity rates, in part due to climatic conditions favorable to the parasite and certain dietary customs, such as the consumption of undercooked red meat or exposure to stray cats [15]. It is crucial, however, to highlight that our 8% prevalence is based on PCR detection of parasitic DNA, which identifies active infections. In contrast, many large‐scale epidemiological surveys, including those in Europe, rely on serology to detect IgG antibodies, which indicates lifetime exposure rather than current infection. Seroprevalence is expectedly higher as it represents a cumulative measure of both past and present infections. This fundamental difference in methodology is a primary reason why our molecular‐based findings are lower than many seroprevalence reports and underscores the difficulty in making direct comparisons [16]. Nonetheless, discrepancies in prevalence between different regions whether within a single country or across geographic boundaries underscore how environmental factors, sanitation measures, sociodemographic differences, and diagnostic methods influence infection rates [10]. In some parts of Iran (for instance, areas with more humid climates or with cultural habits of consuming raw/undercooked meat), higher frequencies of toxoplasmosis have been documented [17–19]. Although only a small fraction (8%) of the women experiencing spontaneous abortion tested positive for *T. gondii*, it is clinically relevant that once primary infection occurs in nonimmune pregnant women, the parasite can cross the placenta and infect the fetus [2, 20]. Indeed, even a relatively small percentage of acute infections among pregnant women can pose major risks for congenital toxoplasmosis and its sequelae, including severe neurological and ocular damage in neonates [6, 20]. The strong, statistically significant correlation in our study between *T. gondii* infection and contact with domestic animals, particularly pet care, underscores the critical role of animal exposure as a primary risk factor. This aligns with meta‐analyses showing elevated odds ratios (OR 1.5–2.8) for toxoplasmosis in individuals with frequent cat exposure (4). Our results strongly suggest that public health messaging should emphasize careful hygiene after contact with pets, especially cats, and their environments (e.g., litter boxes) to mitigate transmission risk [21, 22]. Furthermore, althpugh some investigations have noted that women with spontaneous abortions exhibit higher odds of *T. gondii* antibodies, our data did not show a statistically significant association with previous obstetric history, suggesting that the current infection may be a primary event [8, 23]. Although our data did not show a statistically significant association with previous obstetric history, the possibility remains that undetected or past subclinical infections could contribute to negative pregnancy outcomes [24]. From a clinical standpoint, screening pregnant women for toxoplasmosis especially early in gestation may help identify those at risk of maternal–fetal transmission and allow timely preventive or therapeutic measures [25]. Many authors advocate routine serological or molecular testing, particularly in areas with known high prevalence, to reduce vertical transmission [26]. Since our data indicate that a considerable portion (92%) of the women with spontaneous abortion did not have evidence of infection (i.e., they were presumably susceptible), health education and prophylactic strategies become critical. Simple interventions such as thorough cooking of meats, improved hand hygiene after handling raw meat or soil, and avoiding contact with stray cats could significantly reduce the incidence [10]. An especially noteworthy finding from our study was that all *T. gondii*‐positive cases were detected in pregnancies that had progressed beyond 8 weeks of gestation. This observation may be attributable to several biological factors. First, following a primary maternal infection, it may take several weeks for the parasite load to become high enough in placental tissue to be detectable by PCR. Second, this finding may reflect key changes in placental development. During the first trimester, the placenta is undergoing rapid morphogenesis, and the trophoblastic barrier is still maturing. It has been suggested that the initial robust, multilayered nature of the early trophoblast provides a more effective barrier, whereas the subsequent vascularization and thinning of the placental membrane after this early stage, while essential for fetal growth, might increase its permeability to pathogens like *T. gondii*. Finally, it is also plausible that infections occurring very early in gestation (< 8 weeks) cause catastrophic damage to the developing embryo, leading to a complete failure of implantation or an early abortion that is not captured in a hospital‐based sample, thereby creating a selection bias in the cases available for analysis [27]. The present study′s main limitations include the relatively modest sample size (*n* = 100), which may limit statistical power and generalizability, particularly for rarer variables like pet care (only 4% prevalence). Additionally, reliance on self‐reported data for behaviors such as animal contact introduces potential recall bias, whereas the cross‐sectional design precludes establishing causality between these associations and abortion outcomes. The absence of paired serological testing (e.g., IgM/IgG) means we could not distinguish acute from latent infections, potentially underestimating the parasite′s role. Furthermore, convenience sampling from a single hospital may not fully represent the broader Birjand population, and unmeasured confounders (e.g., detailed dietary habits or stray cat exposure levels) could influence results. To address these, larger scale investigations that incorporate both serological and molecular analyses of maternal and fetal samples could offer more definitive insights into the role of *T. gondii* in spontaneous abortion. Likewise, collecting more robust environmental and nutritional data would help elucidate the true risk factors underlying infection. Longitudinal (cohort) research following women from early pregnancy through delivery might also help in clarifying how and when *T. gondii* exerts its pathological effects, and whether prophylaxis or early detection could mitigate poor outcomes. Overall, our finding that 8% of placental samples were positive for *T. gondii* DNA suggests that this parasite may be a relevant factor in a subset of spontaneous abortions in this region. Given the limitations of this study, including the small sample size and the absence of serological data to confirm acute infection, these results should be interpreted with caution. Strengthening public awareness of transmission routes and improving diagnostic vigilance could be valuable components of prenatal care. To better clarify the parasite′s role and confirm these preliminary findings, larger scale studies that integrate both molecular and serological methods are essential. Such research would help establish causality and pave the way for more targeted health education and screening policies to reduce morbidity associated with *T. gondii* infections in pregnancy.

## Author Contributions

All authors contributed to the study conception and design. Material preparation, sample collection and analysis were performed by Peyman Baghezadeh, Amir Tavakoli Kareshk, Mahmoodreza Behravan, and Davod Javanmard. The first draft of the manuscript was written by Amir Tavakoli Kareshk and Seyed Mohammad Mousavi; and all authors commented on previous versions of the manuscript.

## Funding

No funding was received for this manuscript.

## Disclosure

All authors read and approved the final manuscript.

## Ethics Statement

This study was approved by Birjand University of Medical Sciences (Birjand, Iran) (Ethics Committee Approval ID: IR.BUMS.REC.1402.208).

## Conflicts of Interest

The authors declare no conflicts of interest.

## Data Availability

Research data are not shared.
